# Providing Intensive Palliative Care on an Inpatient Unit: A Full-Time Job

**DOI:** 10.6004/jadpro.2016.7.1.4

**Published:** 2016-01-01

**Authors:** Linda Drury, Kate Baccari, Amy Fang, Courtney Moller, Ian Nagus

**Affiliations:** Dana-Farber Cancer Institute/Brigham and Women’s Hospital, Palliative Care Unit, Boston, Massachusetts

Almost daily, we are asked "How do you do this every day?" by our colleagues, our patients, and their family members. They often associate palliative care with poor patient prognosis and hospice, and they associate hospice with imminent death. During a routine workday, we also come up against many assumptions: "Palliative care? That must be easy. You don’t do anything." "The patient is on the palliative care team. So why are you calling us to do a procedure?"

In our work, we are called at times to pronounce death for some of our patients and to arrange hospice at home or in facilities for others. During almost every shift that we work, we see patients who have come in with a heavy symptom burden. But with good medical management, we have been able to alleviate the symptoms, allowing the patient to return home and continue with his or her medical treatments, work, and important life events.

The answer to the opening question of this article is simple. Most of us enter the field of medicine because we want to help people. During our education and work experience, we quickly realize that medicine has become compartmentalized and subspecialized, limiting our clinical scope to a specific organ system, procedure, or problem.

Those of us who work in the palliative care setting have found a place that encourages us to sit with patients, learn what their goals are, look at the "big picture," and help them and their family navigate the medical system to make sure it works for them, not on them. We allow ourselves time for trial and error in symptom management and space for candid conversations, including about fears of the unknown. Because of this, we believe that we make a difference and help our patients and their families find comfort and clarity in times of medical challenges and even crisis.

## WHAT IS PALLIATIVE CARE?

Although palliative care only became a Board-certified subspecialty of internal medicine in the United States in 2006, it has existed in some form for thousands of years. The concept of palliative care is intuitive and ancient: People have watched over their loved ones; sat vigil beside the dying; and employed herbs, prayers, and talismans to try to relieve suffering over the ages.

Cicely Saunders, a registered nurse, social worker, and a physician, is credited with starting the modern hospice movement when she founded St. Christopher’s Hospice in London in 1967. With her patient care, teaching, and research, she helped establish the field of palliative care worldwide. Now, the number of hospitals in the United States with 50 beds or more that have a palliative care service is growing, with more than 1,700 in 2012 (Center to Advance Palliative Care, 2012).

How is palliative care different from just "good medical care"? Traditionally, a patient with a progressive disease has had to choose between options that can appear from the outside to be a choice between "doing everything" or "doing nothing." Only after curative options had been exhausted was more attention been paid to discussions of overall goals of care, "bucket lists," or more in-depth discussions about choices between procedures or treatments with changing risk/benefit ratios.

Palliative care emphasizes a holistic approach to patients, with goals of relieving not just physical suffering, but also giving attention to the emotional and spiritual needs of patients and their caregivers. Our focus is quality of life, helping patients to live as well as possible for as long as possible in the setting of advanced illness. We do this by learning from patients which symptoms are most bothersome, what their goals and wishes are, and how best to align their care with those goals.

The success of this approach was highlighted in an article that was published in an issue of *The New England Journal of Medicine* in 2010 (Temel et al., 2010). Drs. Jennifer Temel and Joseph Greer conducted a study of palliative care at Massachusetts General Hospital, enrolling patients with lung cancer. All of the patients enrolled were treated with standard-of-care therapies for their cancer, but half of them also received palliative care early in the course of their disease. When they were evaluated at the end of 3 months, the patients on the palliative care arm reported a higher quality of life and less depression, had less aggressive end-of-life care, and lived 2.7 months longer than those patients who did not receive early palliative care (Temel et al., 2010).

## INTENSIVE PALLIATIVE CARE UNIT

Our Intensive Palliative Care Unit (IPCU) is a 12- to 14-bed specialty unit dedicated to the provision of tertiary acute palliative care to patients at Dana-Farber Cancer Institute/Brigham and Women’s Cancer Center (DFCI/BWCC). After an initial pilot program in 2006, this service has served those who need inpatient management, have intense personal or symptomatic distress, or whose families are in severe distress. In comparison to many similar units around the country, our IPCU does not require distinct goals-of-care criteria for admission or transfer. Therefore, oncology patients admitted to our IPCU do not have to have a do-not-resuscitate/do-not-intubate (DNR/DNI) code status and can be undergoing curative-intent therapies. They may be early in their diagnosis or admitted from home hospice for improved symptom management.

In 2009, the IPCU evolved to provide support to those who are eligible for inpatient hospice benefits. Unlike our IPCU patients, these patients do not have to have an oncologic diagnosis, and they must have a code status of DNR/DNI to meet inpatient hospice-eligibility requirements. Typical refractory symptoms for referral to the IPCU include pain, delirium, constipation, nausea/vomiting, diarrhea, dyspnea, and psychosocial distress, among others.

The Figure below shows the percentage of patients admitted to our IPCU with various types of malignancy (from 2012–2013), highlighting the diversity of our patient population.

**Figure 1:**
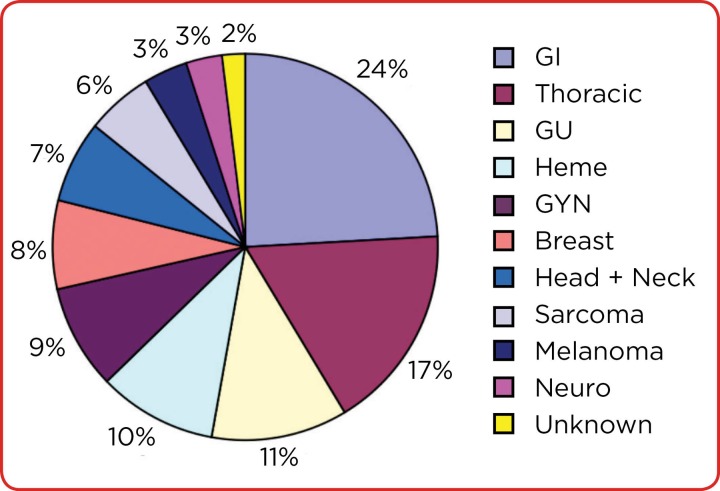
The percentage of patients admitted to our Intensive Palliative Care Unit with various types of malignancy (from 2012–2013), highlighting the diversity of our patient population.

## OUR INTENSIVE PALLIATIVE CARE TEAM

From its inception, the interdisciplinary team model on the unit was designed to optimize comprehensive, cost-effective, and innovative medical care. At DFCI/BWCC, we have an Inpatient Palliative Care Unit, and a separate palliative care consult team.

The consult team consists of attending physicians, several advanced practice nurses, and a social worker, all trained in palliative care, who work alongside the medical teams, who request recommendations for difficult-to-manage symptoms or help in clarifying the goals of care consistent with the wishes of patients and family.

On the IPCU service, physician assistants are the first responders for our patients. All of our patients have advanced disease; most have cancer and are admitted with symptoms caused by their cancer, its treatments, or both. With each presenting symptom, we look for the etiology: interviewing patients, ordering lab tests, and ordering imaging and specialty consults as needed. Medications and treatments are determined based on the patients’ comorbidities; organ function; concurrent medications; and other factors, including the home situation, family support, and mental status.

The IPCU is an interdisciplinary team. It includes five physician assistants, a palliative care–trained attending physician, a dedicated social worker with experience in palliative care, a pharmacist trained in oncology and palliative care, and registered nurses who have chosen to work with our patient population. Our chaplains provide spiritual support, and we have dedicated care coordinators, personal care assistants, unit clerks, and housekeepers who interact daily with patients and their families.

## HOW DO WE PROVIDE PALLIATIVE CARE?

How have we learned to do this work? Like all advanced care practitioners, our initial training in medicine is broad. Once hired to work on the IPCU, we receive specific training in both oncology and palliative medicine; however, the majority of our knowledge comes from day-to-day experiences and interactions. We have come from different backgrounds, not all oncology, and none is from the world of pain management. Through on-the-job training, we have become well versed in the nuances of symptom management and opioid conversions, management of nausea, vomiting, and delirium, symptoms that we encounter daily.

In our pockets, we carry books that are full of tables and opioid conversion charts, peak onsets, and durations of action, but our challenge is to explain all this information to patients and their families in ways they can understand. A greater challenge yet is to listen. Although other specialists practice and perfect procedures, the palliative care "procedure" is the family meeting. It has been noted that the average amount of time a patient is allowed to speak before a clinician interrupts is 18 seconds (Beckman & Frankel, 1984). It is difficult to unlearn this need to speak, to hurry along the conversation, to get to the point, and to move on to the next patient. Listening to the patient is crucial to elucidate symptomatology, develop a differential diagnosis, and choose the best options for treatment in alignment with patients’ goals.

We work at this as a team. Sometimes the risks of swallowing another pill for a patient with a high risk of aspiration outweigh the benefit that pill may provide, and in such cases, our pharmacist offers alternatives. Our bedside nurse may tell us of a patient’s fears and worries about what an upcoming procedure or scan might mean. Our social worker may discover that a patient is the primary caregiver for an elderly parent, allowing the team to explore whether undergoing a potentially debilitating surgery is in line with the patient’s broader goals. These discussions are critical in providing good palliative care to our patients.

We serve as each other’s support, as the things we experience during our workdays are difficult to speak of with friends or family. However, within our office walls, we laugh, share our knowledge and experience, and debrief after difficult meetings and sad cases, of which there are many. We rejoice over small victories and commiserate over electronic medical records and bureaucratic red tape.

## FUTURE GROWTH OF ADVANCED PRACTITIONERS IN PALLIATIVE CARE

Since 2006, we have built relationships with our oncologists and other clinicians, who in turn reassure their patients that referral to the palliative care team does mean the end is near. Rather, the clinicians know that we provide excellent, holistic care and symptom management. Through the work that we do, we have been fortunate to serve as educational and cultural leaders throughout the hospital and community, acting as ambassadors for palliative care for our colleagues as well as our patients and their families.

Attention to palliative care will continue to grow. We have an increasingly aging population, limited financial resources, and the advances in medical care and technology make it possible for us to live longer with serious illness. We have many more options for treatment, but often decisions about treatment are made quickly, with a false urgency, without taking time to ascertain patient preferences and provide care concordant with those preferences.

With an increasing number of palliative care programs in hospitals across the country, more and more research is being done on outcomes and costs. In addition to the Temel study previously mentioned (Temel et al., 2010), several studies have suggested that providing palliative care can reduce costs by "matching treatments provided to patient and family-determined goals for medical care" (Smith et al., 2003).

Organizations interested in palliative care are increasingly looking at how to provide quality medical care to more patients and their families. The National Consensus Project is one such umbrella group that includes a broad range of health professionals and organizations; it has put together Clinical Practice Guidelines for Quality Palliative Care, which have been incorporated into JCAHO (Joint Commission on Accreditation of Healthcare Organizations) recommendations, among others (American Academy of Hospice and Palliative Medicine et al., 2004; Joint Commission, 2008). These comprehensive guidelines include background on and theory of palliative care, recommendations from organizational levels to individual levels, as well as timing and coordination of palliative care with conventional care.

In reality, front-line clinicians provide the majority of palliative care today. However, increasingly specialized training and certificate programs are being offered for care providers of all types. Basic palliative care is vital because hospice and palliative medicine specialists will never be sufficient in number to provide regular face-to-face treatment of every person with an advanced serious illness. The services offered by hospice and palliative medicine specialists supplement—but do not replace—the palliative care services of clinicians in primary care and disease-oriented specialties (Institute of Medicine, 2015).

More important, for those who are already working in palliative care, we need to be aware of how we model good palliative care skills and teach by example. Each interaction with a patient or colleague offers us another chance both to teach and to learn how better to care for our patients.

Advanced practice clinicians can fill a major role both in providing palliative care and in educating others. Although palliative medicine and hospice care are increasingly in the forefront of medical planning and legislation, with entities such as the US Senate, the JCAHO, the Institute of Medicine, and the American Society of Clinical Oncology developing statements, policies, and legislation, recommendations listing suggested makeup of interdisciplinary teams have at times not specifically included the role of physician assistants (Institute of Medicine, 2013). We on the IPCU and other physician assistants involved in palliative care are working through our physician assistant constituent associations to involve our input to be able to continue to provide care in these settings.

## OUR BIG PICTURE

Despite the challenges that come with a relatively new and growing specialty, the full-time physician assistants and nurse practitioners of the DFCI/BWCC palliative care team have consciously decided to continue to do this job day in and day out. We have chosen to provide the type of care that we see as ideal and to build what we believe is a unique and therapeutic medical team.

In his book *Being Mortal*, Atul Gawande (2014) writes of some of these challenges as he accompanies his parents and family through his father’s final illness:

*One of the beauties of the old system was that it made these decisions simple. You took the most aggressive treatment available. It wasn’t a decision at all, really, but a default setting. This business of deliberating on your options of figuring out your priorities and working with a doctor to match your treatment to them was exhausting and complicated, particularly when you didn’t have an expert ready to help you parse the unknowns and ambiguities. The pressure remains all in one direction, toward doing more, because the only mistake clinicians seem to fear is doing too little. Most have no appreciation, but equally terrible mistakes are possible in the other direction—that doing too much could be no less devastating to a person’s life.*

In palliative care, we never believe "there is nothing left to do." Cicely Saunders did not accept those words. There is always room for symptom relief, comfort, empathy, and accompanying our patients through their journey. When it looks like there is nothing left to do, palliative care providers have a firm belief that there is indeed so much more we can do.
